# A Cognitive Behavioural Intervention Programme to Improve Psychological Well-Being

**DOI:** 10.3390/ijerph16010080

**Published:** 2018-12-29

**Authors:** Birgitta Ojala, Clas-Håkan Nygård, Heini Huhtala, Philip Bohle, Seppo T. Nikkari

**Affiliations:** 1 Faculty of Social Science, Health Unit, University of Tampere, 33014 Tampere, Finland; Clas-Hakan.Nygard@staff.uta.fi (C.-H.N.); Heini.Huhtala@staff.uta.fi (H.H.); 2 Tullinkulma Occupational Health Unit, 33100 Tampere, Finland; Seppo.Nikkari@staff.uta.fi; 3 Tasmanian School of Business and Economics, University of Tasmania, Private Bag 84 Hobart, Tasmania 7001, Australia; philip.bohle@sydney.edu.au; 4 Faculty of Medicine and Health Technology, Tampere University, 33100 Tampere, Finland

**Keywords:** stress, occupational health, intervention, burnout, well-being

## Abstract

Psychosocial risk factors have increased in today’s work environment, and they threaten work ability. Good workplace atmosphere, psychosocial support, the ability to cope with stress, and skills and knowledge are all connected to more successful coping. Faster changes in the work environment and an increased workload can lead to a chain of fatigue and illness. The aim of this study was to evaluate a cognitive behavioural intervention as an early rehabilitation strategy to improve employees’ well-being, in intervention group N446 and in control group N116. The well-being measures used were the Bergen Burnout Inventory (BBI 15), Utrecht Work Engagement Scale (UWES), and depression and stress screening questions. Data were obtained by a self-report survey at baseline and at a nine-month follow-up. Differences were analysed within and between groups. The results suggest that cognitive behavioural intervention as an early rehabilitation programme will increase employees’ well-being measured by BBI 15, UWES, and depression and stress screening questions. In the intervention group, the total BBI 15 score (*p* < 0.01) and each of the three subdimensions of burnout (exhaustion, cynicism, and sense of inadequacy) decreased at follow-up. Mental health issues are the commonest reasons for sick leave and early retirement. We need ways to prevent these issues.

## 1. Introduction

Work is changing, and so are work-related occupational hazards [1]. Work-related psychosocial factors are considered a new type of occupational hazard. They include work characteristics and demands, overload and mental stress, workers’ opportunities to influence work tasks and procedures, their use of knowledge and skills, and difficulty and hurry at work [1,2].

These factors increasingly influence workers’ capacity to cope at work. A prolonged discrepancy between employees’ capacities and work demands may produce burnout, which consists of three main symptoms: Exhaustion, cynicism, and reduced professional efficiency. Burnout is typically associated with absenteeism, sick leave, job turnover or physical health issues [3,4,5]. Approximately 5% of the Finnish population suffer from depression annually, and there is a reciprocal relationship between burnout and depression symptoms [6,7].

Participatory interventions that focus on the individual as well as on the organisational level have been shown to be effective in treating burnout [8]. Successful intervention programmes against burnout can be enhanced with refresher courses [9]. It is also important to recognise different burnout patterns and to focus activities effectively [10,11,12].

Employee engagement can be built at work through meaningful experiences and by enabling workers to understand why they are doing the work. In the health sector, people usually describe their work as meaningful and valuable. Everybody ascribes meanings to their work—for example, to the nature of the work role, and to the relationships that they build with others—and these have implications for their experiences of work. Employees are usually fully engaged in contexts where a source of meaningfulness is present. Agreeable identities with clear roles, important work relationships, challenging work, supportive leadership, and the pursuit of rewards all increase engagement. Employees’ engagement can thus be improved by supervisors, leaders, human resources staff, and other co-workers. Under these conditions, workers do their best, are loyal to their employer, and are willing to be flexible if the work so requires [13]. It has been shown that the quality of nurses’ work improves with such engagement [14].

Job strain may precipitate clinical depression among employees, according to a review of six studies with a total of 27,461 participants and 914 incident cases of clinical depression [5]. In some organisations, best practices for managing workplace stress have included context-specific interventions, combined organisational and individual interventions, a participative approach, and a change in culture [15]. When office employees were allocated to social and physical environmental intervention groups, social–environmental intervention showed an improvement in task performance, whereas physical environmental intervention showed an improvement in absorption [15]. Workplace-based, high-intensity psychological interventions may improve work disability outcomes for workers with common mental health conditions [16,17]. However, in a meta-analysis of effects of occupational stress management intervention programmes, cognitive behavioural therapy (CBT) interventions consistently produce larger effects than other types of intervention [18].

The CBT model of intervention encourages individuals to act by themselves to achieve their own goals by supporting them to take actions towards those goals [18]. CBT has been found to be effective in improving work-related stress, depression, anxiety, chronic pain, chronic fatigue syndrome, and insomnia. It has also been found to increase work engagement within a working population [19].

Burnout reflects a negative relationship of hostility and alienation between the person and his/her job, the positive opposite of which is engagement, a relationship of reconciliation, and acceptance [20]. We conducted a prospective study to evaluate the effects of a CBT intervention to improve employees’ well-being, as measured by outcome of questionnaires on psychosocial variables from positive and negative directions.

## 2. Material and Methods

### 2.1. Participants

In 2011–2014, our outpatient intervention study recruited a total of 779 municipal employees. Participants were volunteers who met the inclusion criteria for the study: Being employed in the public sector and working as permanent or long-term temporary staff with at least one year of service. The study was a nine-month follow-up designed to study the causal impact of the intervention on an intervention group, with a control group that did not take part in the intervention. Of the 779 total participants, 594 took part in the intervention group and 185 in the control group. Control group members had the opportunity to take part in the intervention after they had answered follow-up questionnaires before the intervention started. The intervention sessions lasted for four months, with one session every two weeks; five months after that came the follow-up tests and group meetings. The intervention was conducted during paid working hours, and participants were required to commit to the entire programme.

Of the 779 participants, 80% were women and 20% were men. The mean age of subjects was 49.9 years (range 21–64 years). There were no statistically significant differences between the intervention group and the control group in age, gender, body mass index, marital status or years of work experience (Table 1). However, there was a difference in education: The intervention group had less vocational training than the control group. The subjects were recruited from different vocational areas for the intervention programme. The largest participation of women came from health services (37.3%), and of men from construction and transport (70.4%) (Table 2).

**Figure 1:**
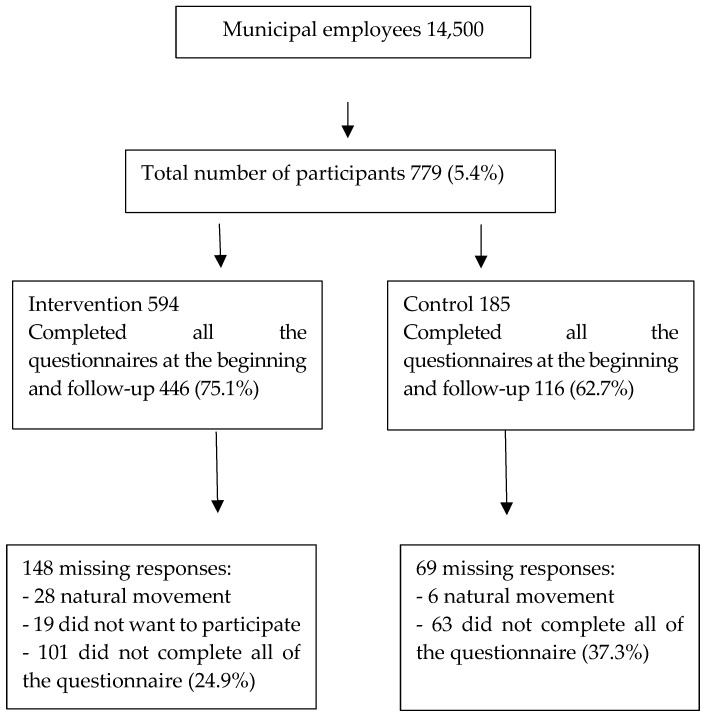
Participants in this study.

In the intervention group, 446 (75.1%) completed the questionnaires at both baseline and follow-up. There were missing responses in 148 cases. In 28 of these, there was natural movement, such as changes of workplace, absence, changes of job, and death. Nineteen cases did not want to take part in the study, and 101 answered incompletely at the baseline or follow-up. In the control group, 116 (62.7%) answered at baseline and follow-up, there was natural movement with six participants, and 63 answered incompletely at baseline or follow-up (Figure 1).

### 2.2. Intervention

An interdisciplinary, goal-oriented multi-professional team (a doctor, an occupational physiotherapist, an occupational psychologist, and a nurse) facilitated each intervention subgroup. The total intervention group was broken down into smaller subgroups for the purposes of the intervention. Goals were set with the participants, who each defined their own goals to improve their work ability. The subgroups met regularly for four months (one day every two weeks). After a further five months, there was the follow-up, which consisted of a three-hour subgroup meeting.

The intervention consisted of different educational components—for example, related to physical training, it was important that all participants understood their own physical test results and how to improve their aerobic condition, muscle strength, balance, and coordination. Physical training included identifying several aspects of one’s physical condition and conducting practices based on those aspects, such as aerobic training focused on one’s pulse level, or training for strength, balance, and coordination. During group reflection, all participants shared their experiences for last two-week period, providing feedback on what have they done to achieve their goals. Work well-being is in direct connection to the work and coping at work. Participants analysed their everyday work-related problems and found ways to understand changes at work and change-related phenomena and they learned new problem-solving skills and skills to talk with their supervisors at work about their work and develop work relationships in everyday life. Work-related problem-solving skills were practised by analysing the elements of one’s work. Skills to talk and develop work relationships in everyday life were practised by starting recommended conversations in the workplace with one’s supervisor concerning one’s work and its daily challenges. Participants had experience on how to set short- and long-term individual goals and what kind of changes are realistic in their life situation.

On each intervention day, there were discussions of the issues affecting participants’ work ability. Every meeting started with individual reflections on the previous two weeks, including things that had been successful, as well as challenging situations. Groups were directed to try to find solutions to challenging situations, rather than to concentrate on problems; to analyse their own work with tools that would help them to see changes in their working lives from a new perspective; to start conversations with their supervisors according to their own interests; and to plan their own paths towards their goals. Peer support was available during the group conversations, which were described as very meaningful by the participants. The group discussions were well received by the participants.

It was considered important that learning should be transferred to everyday health-related activities as soon as possible, to facilitate long-term effects. The rhythm of sessions supported this self-reliance: The sessions were every two weeks, and between sessions, everybody followed their own schedule. This process made it possible to implement practices around all the relevant issues in everyday life. This would be less easy in institutional rehabilitation, where participants usually spend longer periods away from ordinary life situations.

### 2.3. Study

The study was a nine-month trial to estimate the causal impact of the intervention on an intervention group, with a no-treatment control group that did not take part in the intervention. Only data from participants who had responded to all questions during the intervention and follow-up were included in the data analysis. Invitations to participate in the intervention and control groups were sent out to these employees through their workplace management.

Since this intervention was undertaken at an early, pre-clinical stage, there was no need for a medical certificate to take part. The main purpose was to offer an opportunity for intervention to those who needed some support to maintain their own work ability. This approach ensured that the intervention was offered at a time the participants believed was appropriate for them.

Participants were selected for the intervention by occupational health service professionals who had knowledge of the participants’ medical histories, together with the participants’ employers, who were aware of their work demands and workloads. Selection for intervention was thus undertaken collaboratively between occupational health service professionals and the employer. The employer, however, made the final decision as to whether the person could take paid leave from work on the outpatient intervention days. Employers paid the costs of the implementation, and employees took part during paid work hours. Social security paid compensation for the wage costs.

Widely used questionnaires with established reliability and validity were used. Questionnaires were completed at the beginning of the intervention and during follow-up. All questionnaires were administered to the intervention and control groups at the same time: Before the intervention and after nine months, just before the monitoring day.

The study was approved by the ethics committees of the Pirkanmaa Hospital District and the University of Tampere (No: R11068). Written informed consent was obtained from all study participants.

### 2.4. Measurement

The measurement tools used in this study were the Bergen Burnout Inventory (BBI) and the Utrecht Work Engagement Scale (UWES). All measurements were taken at baseline during the information session (autumn 2011); the intervention group completed the measurements at the follow-up test meeting, and the same measurements were taken for the control group at the same time (autumn 2014). The intervention and control subgroups who answered the questionnaires were from the same work units. Each question, including personal information, such as name and social security number, was numbered. The questionnaires were saved in folders, and the folders were archived according to healthcare requirements. The data were stored in a password-protected Excel file, with personal information removed, for statistical tests.

BBI 15 was used to measure burnout. It includes three sub-dimensions: Exhaustion (five items), cynicism (five items), and sense of inadequacy (five items). The internal validity of this test has been previously described [20,21]. The percentiles for age and gender are presented in the manual: Zero to 74 indicates no burnout, 75 to 84 indicates slight burnout, 85 to 94 indicates moderate burnout, and 95 to 100 indicates serious burnout. In this study, we considered only the total sum of BBI 15 and its subdimensions. The BBI 15 measurement can be used in research and occupational health contexts, because BBI 15 has high item–scale reliabilities and good concurrent validity among managers in Finland and Estonia [3,22].

UWES 9 was used to define three dimensions of work engagement: Vigour (three items), dedication (three items), and absorption (three items) [3]. Persons with high vigour scores report high energy, are willing to invest high effort in their work, and display mental resilience while working; persons with high scores on dedication are inspired by their work, see their work as important, and feel pride in their work; persons with high scores on absorption report giving full attention to their work, and the majority find it difficult to detach from work. The UWES assesses a mental state of accomplishment, which is the opposite to burnout [3,22]. Intercorrelations between the three UWES scales exceed 0.65, and the internal consistency of Cronbach’s *α* is equal to the critical value of 0.70 [23,24]. UWES 9 was developed by Schaufeli and Bakker in the Netherlands [24,25].

Two questions were used to screen for depression: (1) “During the last month, have you often been worried, dismal, depressed or hopeless? Answer yes or no”; (2) “During the last month, have you often been worried about experiencing a lack of interest or unwillingness to accomplish things? Answer yes or no”. One or more affirmative answers indicated probable depression [26]. The stress screening question consisted of a single item: “Stress refers to a situation in which a person feels tense, restless, nervous or anxious, or where it is difficult to sleep because of issues constantly on your mind/due to worry. Are you currently experiencing this kind of stress?” The question was answered on a scale from one (not at all) to five (very much) [27].

### 2.5. Data Collection

The primary measurement tools used in this study were quantitative, like the Bergen Burnout Inventory (BBI15) and the Utrecht Work Engagement Scale (UWES). The intervention and control groups who answered these questionnaires were from the same work unit and they had the same criteria for taking part to the intervention. At the time of the first measurements filling, there was not any group division. We tried to randomise these groups, but we did not totally succeed because of working conditions. All measurements were taken at baseline in the information session of the intervention for both groups; the intervention group completed the measurements at the follow-up test meeting, and the same measurements were posted for the control group at the same time. The questionnaires were distributed to study participants in autumn 2011 and autumn 2014. The participants could fill in the questionnaires during their working hours.

### 2.6. Statistical Analyses

Differences between the groups at baseline were tested using the Mann–Whitney U test or chi-square test for categorical variables. Within-group comparisons between baseline and nine-month follow-up scores were performed using the Wilcoxon signed-ranks test. The main effects and interactions for the scores of the intervention and control groups at baseline and follow-up were tested using repeated measures analysis of variance. *p* Values of less than 0.05 were considered statistically significant. The data analysis was performed with SPSS 23.0 software (IBM Corporation, Armonk, NY, USA).

## 3. Results

The results contain only those answers where all items had been filled in at the beginning and end of the study. Baseline, follow-up, and the changes between baseline values and follow-up for the intervention and control groups are shown in Table 3 for the BBI 15 and UWES.

Total BBI 15 values for the intervention group were 36.9 (standard deviation (SD) 11.8) at baseline and 33.9 (SD 12.3) at follow-up. The change from baseline was −3.0 (*p* < 0.001). Values for the control group were 37.6 (SD 12.2) at baseline and 37.5 (SD 14.4) at follow-up. The change from baseline was 0.1 (*p* = 0.912). The difference in changes between groups was statistically significant (*p* = 0.023).

In the intervention group, the total BBI 15 score (*p* < 0.01) and each of the three subdimensions of burnout (exhaustion, cynicism, and sense of inadequacy) decreased at follow-up. There was no corresponding decrease in BBI 15 scores for the control group. The difference in changes between groups in BBI 15 sub-scores was statistically significant for exhaustion (*p* < 0.001), but not for cynicism (*p* = 0.927) or sense of inadequacy (*p* = 0.016).

Total UWES 9 values for the intervention group were 4.3 (SD 1.1) at baseline and 4.5 (SD 1.1) at follow-up (*p* < 0.001). Values for the control group were 4.2 (SD 1.0) at baseline and 4.4 (SD 1.1) at follow-up (*p* = 0.142). There was no difference in changes (0.2) between the groups (*p* = 0.711), although the change in *p*-value was significant in the intervention group (*N* = 446) compared with the control group (*N* = 116). The total UWES 9 score and all three of its dimensions of work engagement improved in the intervention group (*p* < 0.001).

There was also a similar improvement in total UWES scores and two of its dimensions (vigour and absorption) compared with the control group (0.2), change in dedication in the intervention group was also 0.2, and in the control group, 0.1. It is possible that the questionnaire itself acted as intervention and led to some positive change. However, there were no statistically significant differences in the changes in any UWES scores from baseline to follow-up between the groups, because the change from baseline was very similar in both groups (Table 3).

The composite score for the two depression screening items decreased significantly from baseline to follow-up for the intervention group (*N* = 451), in which 6.4% of scores increased, 13.3% decreased, and 80.3% were at the same level as baseline (*p* = 0.001). There was no significant change for the control group (*N* = 115), in which 12.2% of scores increased, 7.8% decreased, and 80% were at the same level as baseline (*p* = 0.405).

The composite score for the one stress screening question compared with the baseline showed significant differences in the follow-up of the intervention group (*N* = 445): 39% increased, 15.5% decreased, and 45.5% were at the same level as baseline (*p* < 0.001). In the control group (*N* = 117), 24% increased, 26% decreased, and 50% were at the same level as baseline (*p* = 0.596).

## 4. Discussion

The principal finding of this study is a statistically significant improvement in several measures of psychosocial well-being (BBI 15, UWES, stress, depression) for participants who completed the cognitive behavioural intervention programme. No corresponding changes were identified in the control group. There was a significantly greater change in BBI 15 from baseline for the intervention group than for the control group. The UWES questionnaires seemed to produce nearly the same improvement in both the intervention and control groups, although the improvement in the control group was not statistically significant because of the group size. Factors associated with social processes at work seem to be crucial to burnout as measured by BBI 15. Burnout is connected to job demands, a lack of job resources, and health problems. When intervention leads to positive changes in participants’ physical condition or work environment, participants have been shown to be able to modify their self-perceptions, resulting in psychological and behavioural changes, such as increased self-approval, self-mercy, and recognition of their inner needs and limits [28,29,30].

It seems that the effects of our cognitive behavioural intervention to improve employees’ well-being was able to meet some challenges in the improvement of attitudes as measured by BBI 15. The UWES 9, used to define three dimensions of work engagement, showed significant improvement in the intervention group, for whom goals were set in collaboration with the participants, and every participant defined their own goals to improve their work ability. An earlier study also suggested that focusing on work engagement might benefit the individual. Employees who seem to perform better have elevated levels of energy and identification with their work [29].

All three UWES dimensions were at average levels at the beginning and follow-up, although absorption increased to an elevated level in both the intervention and control groups. It may be that the questionnaire acted as an intervention for both groups, regardless of other interventions [30,31].

The composite of two depression screening items showed significant improvement at follow-up for the intervention group. This result contrasts somewhat with earlier evidence that the use of screening for depression is associated with only a modest increase in its recognition. If used alone, screening questionnaires for depression appear to have little or no impact on the management of depression [25]. However, our intervention was performed after initial screening and appeared to influence depressive thoughts positively. The stress screening consisted of a single question, and stress was lower after the intervention. This result is in line with previous findings that cognitive behavioural stress management interventions are more effective than other intervention types [32].

The practical point of intervention is to be aware of the different profiles among employees regarding adjustments in the work and non-work demands they face. It is important to create interventions to support work cultures for diverse ways of working, because there is no single optimal way to manage boundaries between work and non-work. Person-oriented interventions that are tailored to support different profiles are needed [33].

### Limitations of the Study

One limitation is that the participants represent a relatively small population in Finland. The intervention and control groups were selected partly according to the participants’ own interests. The overall workload of every employee in the workplace was considered during the selection process by the employer. This selection may have produced differences between the groups at baseline, and selection for the intervention might be one driver of some changes in the scores. Supervisors played a key role in allocating participants to groups.

A randomised control group could not be used for the intervention because of workplace constraints. Issues that needed to be considered included the timetable of the entire process, holidays, individuals’ work situations, and the need to achieve a sufficient number of participants in the intervention group—there were not the same numbers of participants in the control group. However, the control group was from the same work unit as the intervention group, and participants were chosen as randomly as possible from that environment.

Question-based research may suffer from bias if the participants feel satisfied with the service and therefore respond positively when they answer the second time. Two dimensions of the UWES 9 results also improved in the control group, and statistically, the same change was significant in the larger intervention group, but not in the smaller control group; the change between the groups showed no statistical difference. This kind of long-lasting service includes many changing variables, which makes it difficult to define the causes of the results. A third measurement point would have enabled broader statistical analysis.

In this study, we had a respectable amount of data to ensure its adequacy for possible dropouts. Dropout is a prevalent complication in the analysis of data from follow-up studies, but in this study, there were no differences between those who responded compared with those who did not in terms of age, gender, years of work or work unit. 

As part of our results suggest that the cognitive behavioural intervention was effective in increasing employees’ well-being, we currently have no measures to show its financial benefits to the employer. One recent systematic review has found that it is difficult to draw conclusions about the cost-effectiveness of intervention outcomes, because of the shifting quality of the studies [33].

## 5. Conclusions

This study suggests that a cognitive behavioural intervention achieved significant improvements in several measures of mental health. The results imply that this kind of intervention is needed to give early support on mental health issues for the working-age population. Early rehabilitation allows participants to play an active role while they still have the resources to make changes in their own lives. Overall, the results of this study permit the conclusion that this kind of service does support working ability in today’s municipal sector. It is important to act preventively while participants have the resources to play an active role. Peer support also has remarkable value for finding solutions in different life situations.

## Figures and Tables

**Table 1:** Background characteristics of study population.

	*N*	Intervention	*N*	Control	*p*
Age (years)	578	49.2 (7.8)	173	48.1	0.205
Gender, female (%)	463	80.1	138	79.8	1
Married (%)	578	56.6	173	61.5	1
Years of professional experience	547	19.2 (10.1)	165	18.2 (10.6)	0.702
Education					
No vocational training (%)	38	7.1	9	5.3	0.61
Vocational school (%)	344	64.3	108	63.9	
University of applied science (%)	55	10.3	25	14.8	
University degree (%)	98	18.3	27	16	
Total	535	100	169	100	

**Table 2:** Main occupations of study population.

	Intervention				Control			
	Female	%	Male	%	Female	%	Male	%
	*N*		*N*		*N*		*N*	
Health service	173	37.3	0	0	53	38.4	0	0
Construction and transport	0	0	81	70.4	0	0	22	62.9
Education and day care	69	14.9	9	7.8	22	15.9	4	11.4
Other services	68	14.7	0	0	18	13.1	0	0
Food services	66	14.3	0	0	21	15.2	0	0
Office work	48	10.4	7	6.1	18	13.1	0	0
Management specialist	39	8.4	18	15.7	6	4.3	9	25.7
Total	463	100	115	100	138	100	35	100

**Table 3:** Intervention and control groups at baseline and follow-up on total Bergen Burnout Inventory (BBI) 15 and Utrecht Work Engagement Scale (UWES) and all items, and changes in BBI 15 and UWES, related factors.

BBI 15		Baseline		Follow-Up		Change from Baseline	*p*-Value	Difference in Changes between Groups
	Intervention Group *N* = 425, Control Group *N* = 109	Mean	SD	Mean	SD			
Total BBI 15	Intervention	36.9	11.8	33.9	12.3	−3	<0.001	0.023
	Control	37.6	12.2	37.5	14.4	0.1	0.912	
Exhaustion (5 items)	Intervention	13.2	4.8	12.1	5.2	−1.1	<0.001	<0.001
	Control	12.9	4.6	13.1	5.3	0.2	0.477	
Cynicism (5 items)	Intervention	10.6	4	10	4	−0.6	<0.001	0.927
	Control	11.2	4.2	11	5.1	0.2	0.622	
Sense of inadequacy (5 items)	Intervention	13.1	4.8	11.8	4.9	−1.3	<0.001	0.016
	Control	13.6	4.9	13.4	5.5	0.2	0.68	
**UWES 9**		**Baseline**		**Follow-Up**		**Change from Baseline**	***p*-Value**	**Difference in Changes between Groups**
	**Intervention Group *N* = 446, Control Group *N* = 116**	**Mean**	**SD**	**Mean**	**SD**			
Total UWES 9	Intervention	4.3	1.1	4.5	1.1	0.2	<0.001	0.711
	Control	4.2	1	4.4	1.1	0.2	0.142	
Vigour (3 items)	Intervention	4.3	1	4.5	1	0.2	<0.001	0.555
	Control	4.2	1	4.4	1	0.2	0.154	
Dedication (3 items)	Intervention	4.4	1.1	4.6	1.1	0.2	<0.001	0.919
	Control	4.4	1.1	4.5	1.1	0.1	0.054	
Absorption (3 items)	Intervention	4.1	1.1	4.3	1.1	0.2	<0.001	0.659
	Control	4.1	1	4.3	1.1	0.2	0.232	

Notes: Within-group changes in intervention and control groups after nine months (2) were compared with baseline (1). Difference in changes between groups measured by analysis of variance. Table shows only answers where all items were filled in at the beginning and end of the study.
